# A Ribosome-Related Prognostic Signature of Breast Cancer Subtypes Based on Changes in Breast Cancer Patients’ Immunological Activity

**DOI:** 10.3390/medicina59030424

**Published:** 2023-02-21

**Authors:** Tiankuo Luan, Daqiang Song, Jiazhou Liu, Yuxian Wei, Rui Feng, Xiaoyu Wang, Lin Gan, Jingyuan Wan, Huiying Fang, Hongzhong Li, Xia Gong

**Affiliations:** 1 Department of Anatomy, Chongqing Medical University, Chongqing 400016, China; 2 Chongqing Key Laboratory of Molecular Oncology and Epigenetics, The First Affiliated Hospital of Chongqing Medical University, Chongqing 400016, China; 3 Department of Breast and Thyroid Surgery, The First Affiliated Hospital of Chongqing Medical University, Chongqing 400016, China; 4 Department of General Surgery, Chongqing University Fuling Hospital, Chongqing 408000, China; 5 Chongqing Key Laboratory of Biochemistry and Molecular Pharmacology, Department of Pharmacology, Chongqing Medical University, Chongqing 400016, China; 6 Department of Breast Diseases, Chongqing University Cancer Hospital, Chongqing 400030, China

**Keywords:** breast cancer, immunologic and hallmark gene sets, ribosome related genes, bioinformatic analysis

## Abstract

*Background and Objectives*. The prognostic role of adjacent nontumor tissue in patients with breast cancer (BC) is still unclear. The activity changes in immunologic and hallmark gene sets in normal tissues adjacent to BC may play a crucial role in predicting the prognosis of BC patients. The aim of this study was to identify BC subtypes and ribosome-associated prognostic genes based on activity changes of immunologic and hallmark gene sets in tumor and adjacent nontumor tissues to improve patient prognosis. *Materials and Methods*. Gene set variation analysis (GSVA) was applied to assess immunoreactivity changes in the overall sample and three immune-related BC subtypes were identified by non-negative matrix factorization (NMF). KEGG (Kyoto Encyclopedia of Genes and Genomes) and GO (Gene Ontology) analyses were after determining the prognostic gene set using the least absolute shrinkage and selection operator (LASSO) method. Ribosome-related genes were identified by PPI (protein-protein interaction) analysis, and finally a prognostic risk model was constructed based on the expression of five ribosomal genes (*RPS18*, *RPL11*, *PRLP1*, *RPL27A*, and *RPL38*). *Results*. A comprehensive analysis of immune and marker genomic activity changes in normal breast tissue and BC tissue identified three immune-related BC subtypes. BC subtype 1 has the best prognosis, and subtype 3 has the worst overall survival rate. We identified a prognostic gene set in nontumor tissue by the least absolute shrinkage and selection operator (LASSO) method. We found that the results of both KEGG and GO analyses were indistinguishable from those of ribosome-associated genes. Finally, we determined that genes associated with ribosomes exhibit potential as a reliable predictor of overall survival in breast cancer patients. *Conclusions*. Our research provides an important guidance for the treatment of BC. After a mastectomy, the changes in gene set activity of both BC tissues and the nontumor tissues adjacent to it should be thoroughly evaluated, with special attention to changes in ribosome-related genes in the nontumor tissues.

## 1. Introduction

BC (breast cancer) is one of the most common cancers for women in the world. Moreover, the incidence rate of BC accounts for 30% of all cancers in women, and the mortality rate is as high as 15% [1]. Metastasis in BC patients is the major reason for death. The five-year survival rate is only 25% for patients with metastatic BC [2]. Unfortunately, even after treatment, patients with primary breast cancer still have a 20–30% probability of metastasis [3]. Mastectomy is the main method for early treatment of BC, but BC will still recur after treatment [4]. Despite surgical intervention, there remains a risk of recurrence. Moreover, BC has a high degree of heterogeneity, which leads to the invasion and metastasis of breast cancer to a large extent, making the treatment of BC more difficult [5].

Most previous studies on BC subtypes have focused on pyroptosis-related genes [6], subtypes based on multi-omics and transcriptional patterns [7], and subtypes based on biological and immune components [8]. These studies all provide important clinical implications for the treatment of BC. Nevertheless, few studies have identified BC subtypes by systematically analyzing nontumor tissues adjacent to BC and BC samples for changes in the activity of immunological and signature gene sets.

In this study, we established three different BC subtypes by systematic immunological and marker gene set activity change analysis based on normal breast samples and BC samples. Three clinical subtypes of BC were used to identify a set of prognostic genes in non-tumor samples. Immune cell abundance, immune score, stromal score, estimate score, and tumor purity were evaluated for the three clinical subtypes. KEGG and GO analysis and PPI co-expression network results showed that they were intimately associated with ribosome. Therefore, based on the above results, we further analyzed the specific ribosomal molecular characteristics in BC subtypes, and constructed a risk model according to ribosome-correlated genes to evaluate the survival rate of BC patients.

Our study offers a novel approach for categorizing subtypes of BC and crucial support for BC treatment. It highlights the need to closely monitor the alteration of ribosome-related gene activity in non-tumor tissues surrounding mastectomy sites in BC patients.

## 2. Materials and Methods

### 2.1. Data Collection

RNA-seq data from 1222 BC samples, including 1109 tumor samples and 113 normal samples, and their clinical information were downloaded from the TCGA database (https://portal.gdc.cancer.gov/ (accessed on 24 November 2021)). Gene expression profiling data and associated clinical information for BC were downloaded from the GEO (https://www.ncbi.nlm.nih.gov/geo/ (accessed on 24 November 2021)), including (GSE20685, *n* = 327), (GSE88770, *n* = 116). Gene set enrichment analysis (GSEA) (https://www.gsea-msigdb.org/gsea/msigdb/index.jsp (accessed on 24 November 2021)) software was used to download the 50 hallmark genes sets and 4872 ImmuneSigDB gene sets of C7 [9,10].

### 2.2. Gene Set Variation Analysis (GSVA)

R Package GSVA was utilized to enrich gene sets of TCGA and GEO BC patients’ gene expression profiling data and further observe the changes of gene set activity [11].

### 2.3. Heatmaps and Nonnegative Matrix Factorization (NMF)

The R package “pheatmap” was used to draw heatmaps [12]. TCGA and GEO gene expression profiling data of BC were analyzed using the NMF package [13].

### 2.4. Classification of BC Samples

TCGA and GEO gene expression profiling data of BC were analyzed using the “Cancer subtypes” package [14].

### 2.5. ESTIMATE and ssGSEA Analysis of BC Subtypes

The analysis of 29 immune pathways in a single ssGSEA sample from three subtypes of BC was conducted. ESTIMATE was used to analyze immune cells (immune score), stromal cells (stromal score), tumor purity, and ESTIMATE score for three subtypes of BC [15]. Finally, it is presented with a violin plot.

### 2.6. Construction and Validation of Potential Prognostic Gene Sets Models

The potential prognostic gene sets were identified by the least absolute shrinkage and selection operator (LASSO) method [16]. The prognostic model’s accuracy and OS analysis in BC patients were analyzed using the ROC and Kaplan–Meier algorithms.

### 2.7. Estimation of Tumor-Infiltrating Immune Cells (TICs)

We calculated the proportion of 23 kinds of immune cells in BC patients with the ssGSEA algorithm.

### 2.8. Gene Set Enrichment Analysis (GSEA)

GSEA was performed between high and low group of risk score. Risk score = −0.088 × (RPS18 expression) + −0.155 × (RPL11 expression) + −0.024 × (RPLP1 expression) + −0.153 × (RPL27A expression) + −0.047 × (RPL38 expression). The number of random sample permutations was set at 1000, and NOM *p* < 0.05, FDR q < 0.05, and |NES| > 1 were set as the significance thresholds.

### 2.9. Functional Analysis of GO and KEGG

Gene ontology (GO) and the Kyoto Encyclopedia of Genes and Genomes (KEGG) enrichment analysis of genes in prognosis-associated gene sets were used to clarify their mechanism in prognosis.

### 2.10. String and Protein-Protein Interaction Analysis

Constructing a prognostic gene set protein relationship network through the string data base (https://cn.string-db.org/ (accessed on 24 December 2021)) [17]. The MCODE (Molecular Complex Detection) plug-in Cytoscape_v3.9.0 (San Diego, CA, USA) was used to filter out hub genes [18].

### 2.11. Statistical Analysis

All statistical analyses in this article were performed with R software (Auckland, New Zealand, version 4.1.2, https://www.r-project.org/ (accessed on 24 November 2021)). Statistical differences between two or three groups were compared using the Wilcoxon test. *p* < 0.05 = “*”, *p* < 0.01 = “**”, and *p* < 0.001 = “***”. *p* values < 0.05 would be statistically significant.

## 3. Results

### 3.1. Three BC Subtypes Based on Changes in Activity of Immune and Hallmark Gene Sets

**Figure 1:**
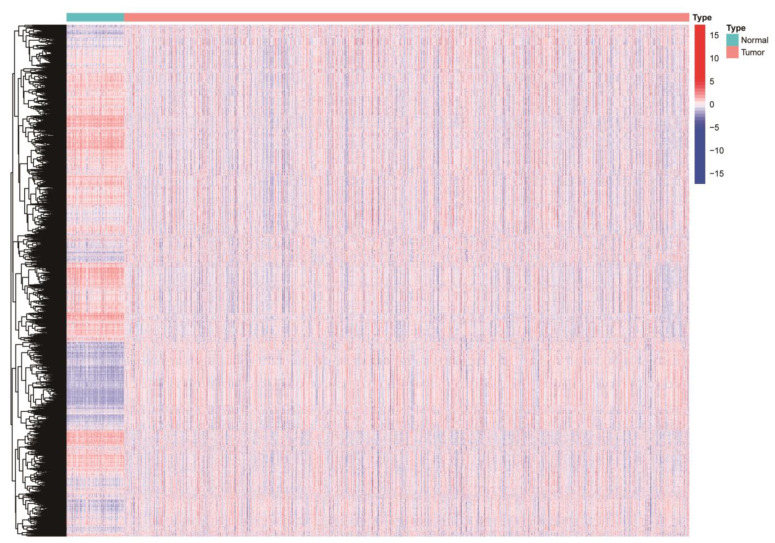
Heatmap of enrichment scores from 4922 immunologic and hallmark gene sets in tumor and non-tumor tissues based on TCGA−BC. N, normal; T, tumor.

**Figure 2:**
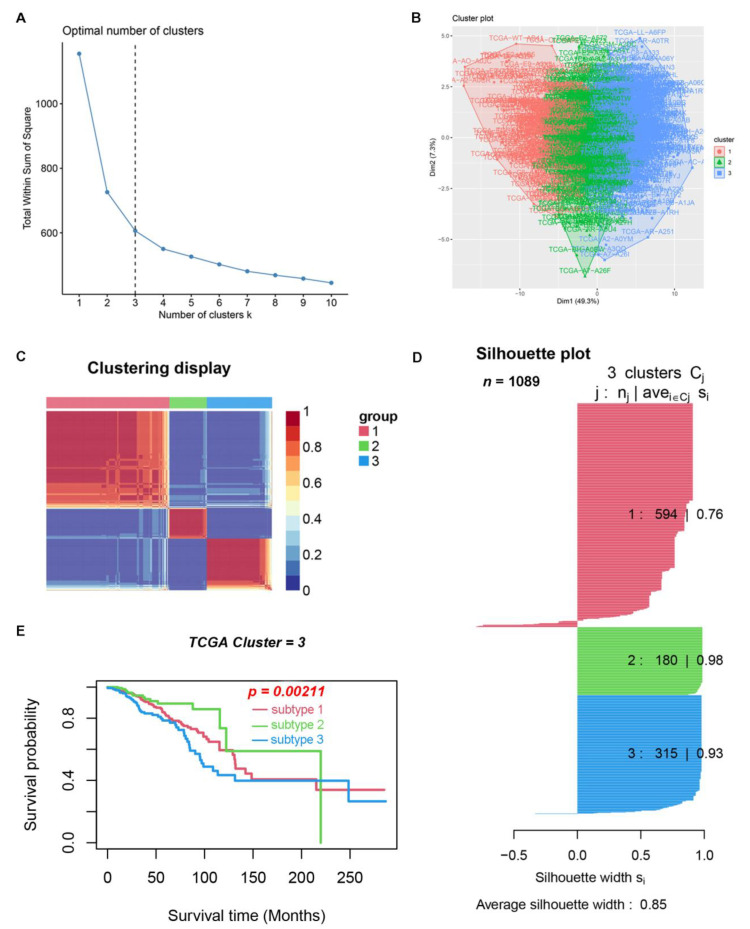
Identification of clinical subtypes associated with BC in TCGA. (**A**) Third, the optimal number of clusters. (**B**) Visualize the cluster results. (**C**) Nonnegative matrix decomposition (NMF) was used for cluster analysis of BC samples. (**D**) Results of the silhouette width plots. (**E**) Kaplan–Meier survival analysis of three BC subtypes.

Gene set variation analysis (GSVA) can be used to detect changes in the activity expression of gene sets in different pathways [11]. To get a comprehensive view of the changes in immune and hallmark sets in breast cancer tissue and nontumor tissue adjacent to BC, we downloaded 50 hallmark gene sets and 4872 ImmuneSigDB gene sets of C7 from gene set enrichment analysis (GSEA) (https://www.gsea-msigdb.org/gsea/msigdb/index.jsp (accessed on 1 October 2021)). After performing GSVA enrichment analysis on TCGA data samples, we found some differences among BC tumor tissues and BC normal tissues (Figure 1). A Cox regression algorithm was developed using the cancer subtypes package for expression level and clinical data to screen 41 clinically related gene sets from 4922 gene sets. The factoextra package was used to screen the optimal K value, which was 3 (Figure 2A,B). The NbClust package was used to classify breast cancer patients for subtypes, and finally three different subtypes were obtained (Figure 2C). The average silhouette width was 0.85 (Figure 2D), indicating the rationality of our BC subtype classification. Patients with BC subtype 2 had the best survival rate, followed by subtype 1 patients and subtype 3 patients (Figure 2E). We used the same method to combine the GSE20685 and GSE88770 data and removed batch effects for the analysis. This result was consistent with TCGA data analysis, that patients with BC subtype 2 had the best survival rate, followed by subtype 1 patients, and subtype 3 patients had the worst survival rate (Figure S1A–E). Detailed clinical information on the three subtypes is presented in Supplementary Table S1.

**Figure 3:**
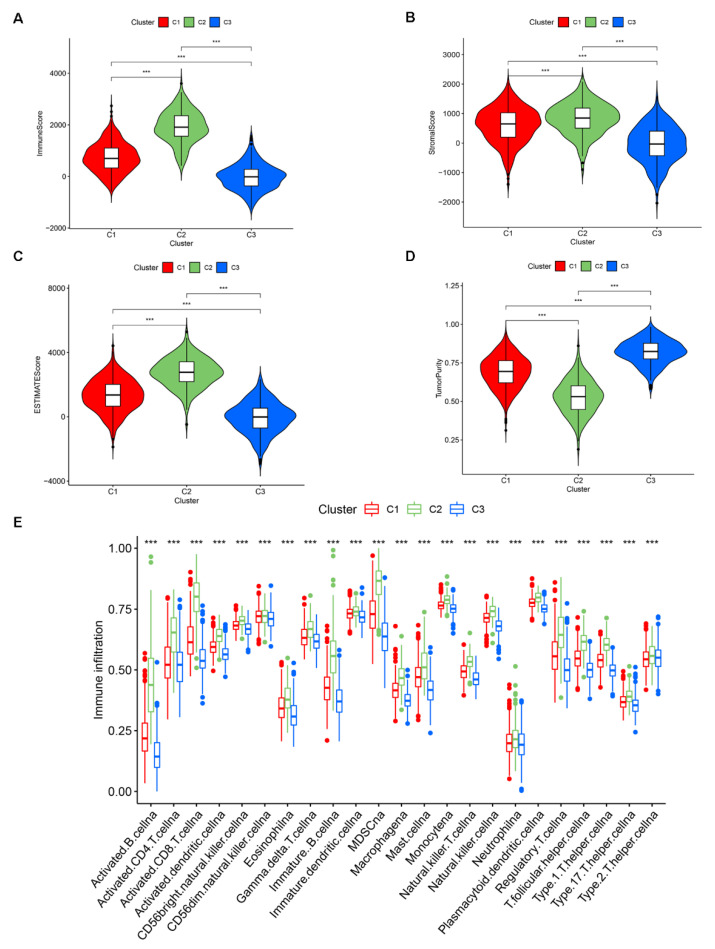
Different phenotypes of BC subtypes in TCGA. (**A**–**D**) The levels of immune cell infiltration, stromal score, ESTIMATE score, and tumor purity were compared among BC subtypes of TCGA. (**E**) Comparison of the expression levels of 23 kinds of immune cells between BC subtypes in TCGA. *p* < 0.001 = “***”.

Recent studies have shown that the tumor microenvironment influences the incidence and development of tumors [19,20], and immune cells and stromal cells interact to play an important role [21]. Therefore, we studied three BC subtypes using the estimate score algorithm. In the TCGA BC patients cohort, the results of three subtypes for the correlation with tumor microenvironment are presented with a violin plot (Figure 3A–C). The results showed that the BC subtype 2 had the highest immune score, stromal score, and estimate score, followed by those of subtype C1, and subtype C3 had the lowest scores. Furthermore, BC subtype C2 had the lowest tumor purity, and subtype C3 had the highest tumor purity (Figure 3D). In the GEO cohort, the BC subtype C2 also had a significantly higher immune score, stromal score, and estimate score than those of the other two subtypes (Figure S2A–C). Additionally, BC subtype C2 had much lowest tumor purity than subtype C1 and subtype C3 (Figure S2D). 

We adopted the ssGSEA method to score 23 kinds of immune cells for the three BC subtypes, and the scores reflected the relational degree of 23 kinds of immune cells for the three BC subtypes. The results showed that subtype C2 had higher scores of immune-related pathways than subtype C1 and subtype C3 in both TCGA (Figure 3E) and GEO (Figure S2E) datasets, and subtype C3 had the lowest scores. The scoring results of both the estimate score algorithm and ssGSEA method (23 kinds of immune cells) remained consistent with our initial survival results obtained for the three BC subtypes. subtype C2 had the best survival, followed by subtype C1 patients, and subtype C3 patients had the worst survival rate, further justifying our BC subtypes classification and illustrating the positive correlation between high immunologic activity and survival.

### 3.2. Analysis on BC Subtypes and Clinical Correlation

**Figure 4:**
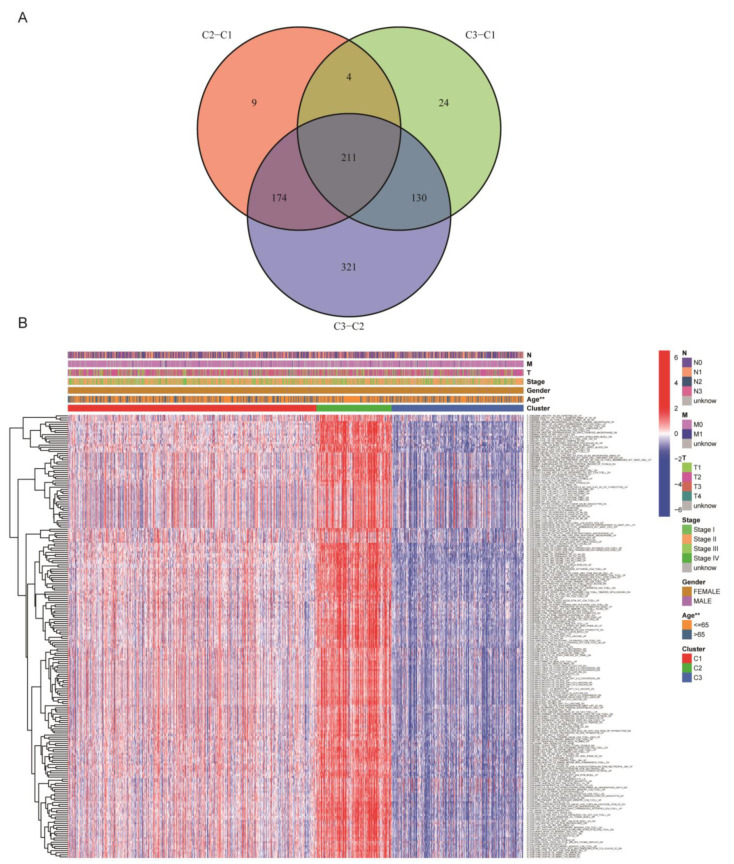
Identification of critical gene sets from BC subtypes. (**A**) A total of 211 differential gene sets were obtained by the differential enrichment score of gene sets between each of the two subtypes and taking intersections. (**B**) Gene sets and clinical associations are shown through heatmap. *p* < 0.01 = “**”.

**Figure 5:**
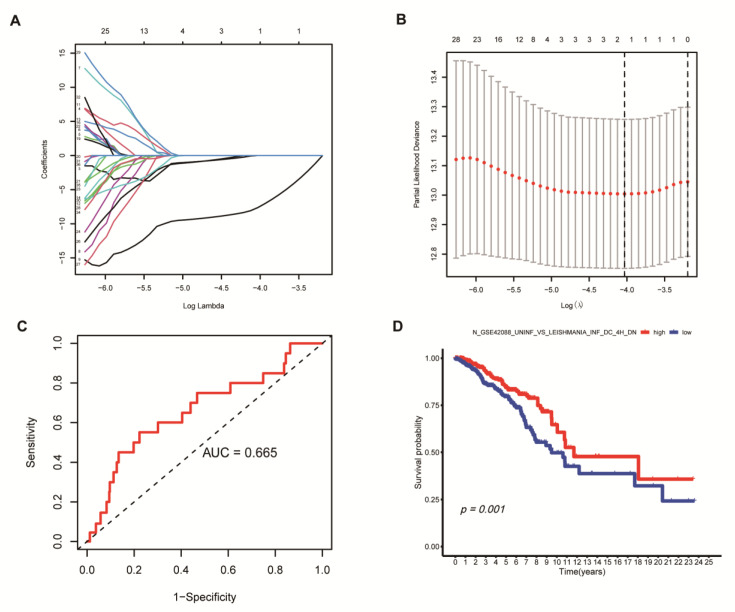
Potential prognostic gene sets in BC (**A**) The most prognostic gene sets were identified by the LASSO algorithm. (**B**) 10 rounds of cross validation were performed to prevent overfitting. (**C**) The ROC curve analysis of gene sets N_GSE42088. (**D**) Kaplan–Meier survival curves of N_GSE42088 gene set.

Next, we analyzed the clinical characteristics of BC subtypes. The results also revealed that patients with BC subtype 2 had the best survival rate, followed by subtype 1 patients, and subtype 3 patients had the worst survival rate in both the TCGA cohort and GEO cohort (Figure 2E and Supplementary Figure S1E). To identify the gene sets that differ among the three subtypes, we first compared the GSVA enrichment scores of normal tissue and tumor tissue to obtain the differential gene sets (adj *p* value < 0.05). logFC > 0 was defined as the T gene set, while logFC < 0 was defined as the N gene set. The differential expression analysis was then performed between each two subtypes, and 211 gene sets that differed between any two subtypes (with the same constraints as above) were obtained. Figure 4A shows 211 representative differential gene sets, and the differential gene sets and clinical correlation are demonstrated with a heatmap (Figure 4B). The heatmap showed that BC subtype C2 had the highest expression level in the differential gene sets, subtype C3 had the lowest expression level, and the three subtypes was highly correlated with age. To further identify the gene sets most related to the prognosis of BC subtypes, we applied the LASSO regression model to finally identify a gene set N_GSE42088 (Figure 5A,B), Risk Score = Coefficients value (−7.63584585568389) × expression of N_GSE42088_UNINF_VS_LEISHMANIA_INF_DC_4H_DN. In Figure 5C, the area under the curve is (AUC) 0.665, proving the reliability of the risk score. The gene set expression level of N_GSE42088_UNINF_VS_LEISHMANIA_INF_DC_4H_DN correlated with the overall survival (OS) of BC patients in TCGA cohort (*p* = 0.001), and patients had a better survival rate when this gene set had a high expression level (Figure 5D).

### 3.3. Functional and Pathway Enrichment Evaluation

**Figure 6:**
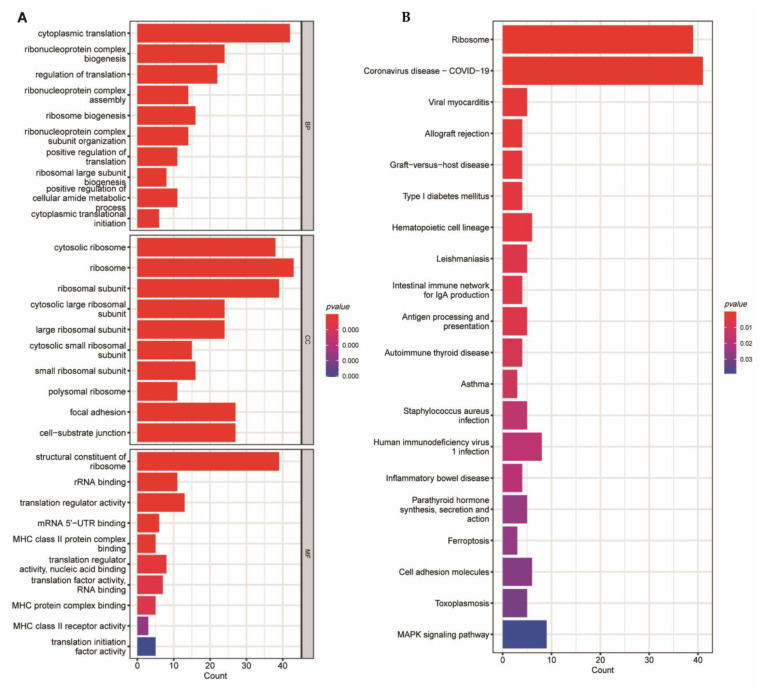
Analysis of GO and KEGG features. (**A**) BP, biological processes; CC, cellular components; MF, molecular functions of enrichment in the N gene set. (**B**) KEGG enrichment results in the N gene set.

**Figure 7:**
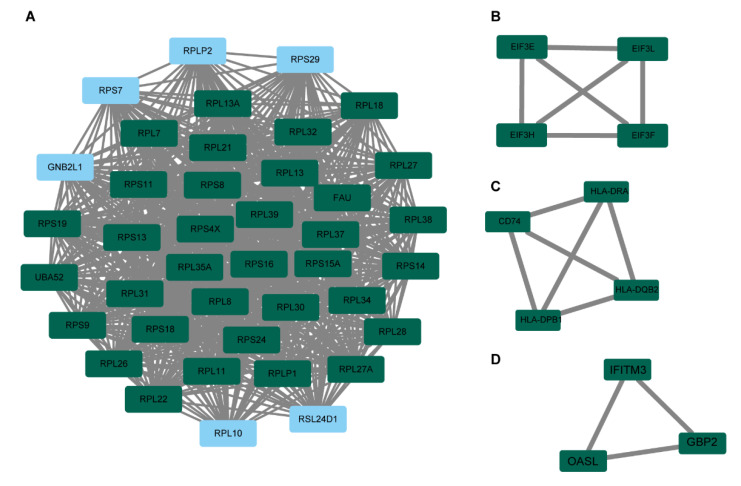
Analysis of protein–protein interaction networks of N gene set using the MCODE algorithm in STRING. (**A**) Cluster 1. (**B**) Cluster 2. (**C**) Cluster 3. (**D**) Cluster 4. The color green indicates the hub genes.

We obtained all the genes in this prognostic gene set and performed KEGG and GO enrichment analysis to reveal the mechanism of its prognosis. The CC (cellular component) of this nontumor gene set was mainly associated with cytoplasmic translation. MF (molecular function) was concerned with ribosome, ribosomal subunit, and cytosolic ribosome. BP (biological process) was connected with structural component of ribosome (Figure 6A). KEGG analysis results were enriched in ribosome and coronavirus disease COVID-19 (Figure 6B). By using protein–protein interaction network analysis, we retained scores greater than 0.9 for subsequent analysis (Figure S3). Intriguingly, the 34 hub genes with the highest degree score in cluster 1 were all associated with ribosomes (Figure 7A), cluster 2 was EIF3E, EIF3F, EIF3H and EIF3L (Figure 7B), cluster 3 was HLA-DRA, HLA-DQB2, HLA-DPB1 and CD74 (Figure 7C), and cluster 4 was IFITM3, GBP2, and OASL (Figure 7D).

### 3.4. Identification of Ribosome-Associated Clusters and Assessment of Immune Cell Infiltration

**Figure 8:**
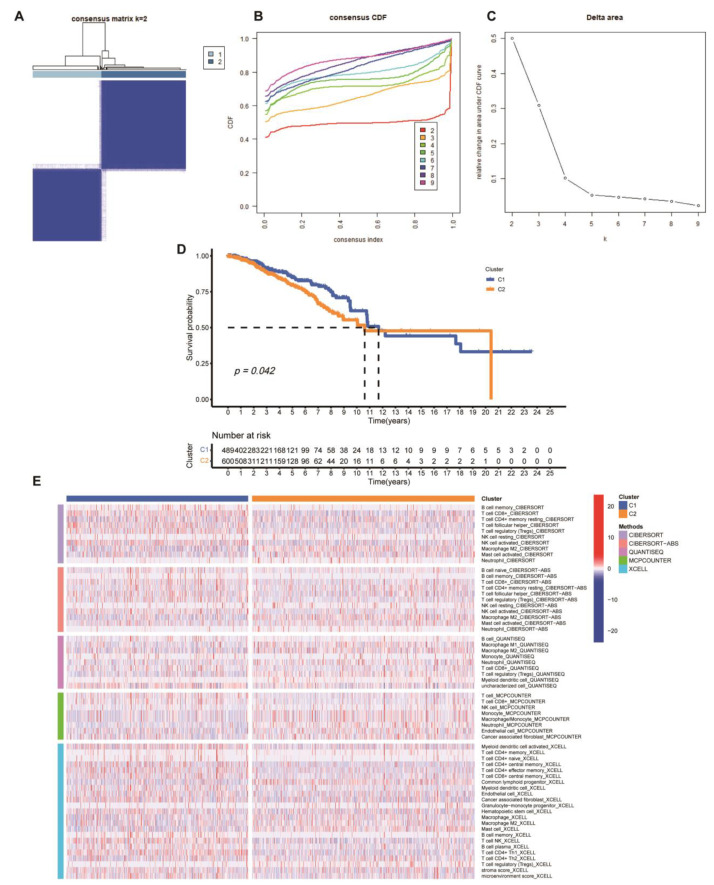
Clustering analysis of ribosome-related genes in TCGA cohort. (**A**–**C**) Identification of ribosome related cluster in the TCGA cohort by NMF method; consensus clustering results K = 2 to 9 were shown, K = 2 was the optimal choice. (**D**) Kaplan–Meier curve survival analysis in different ribosome related clusters. (**E**) Immune cell infiltration in different ribosome-associated clusters.

To explore the role of ribosome related genes in BC, we further used the NMF algorithm to analyze 34 ribosome-related hub genes. In this way, the BC cohort of TCGA was divided into two subtypes with different molecular characteristics and clinical features, including ribosome related subtype C1 and C2 (Figure 8A–C). We found that C1 patients had a notable higher survival rate than C2 (Figure 8D). We used multiple algorithms, including CIBERSORT, CIBERSORT-ABS, QUANTISEQ, MCPCOUNTER and XCELL, to analyze the two subtypes of immunocyte infiltration. The heatmap showed that the proportion of CD8^+^ T cells in C1 subtype was higher than that in C2, while the proportion of Macrophage M2 in C1 subtype was lower than that in C2 (Figure 8E).

### 3.5. Development and Validation of a Ribosome-Related Prognostic Risk Model

**Figure 9:**
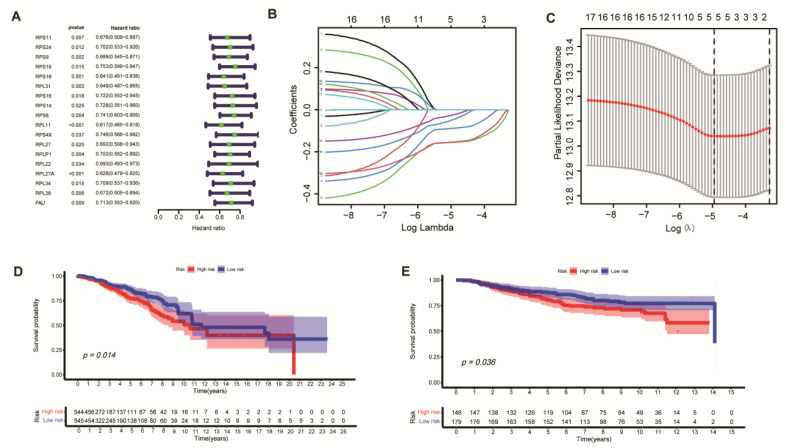
Development and validation of the ribosome-related signature (RRS) based on ribosome-associated clusters in TCGA cohort. (**A**) Univariate COX regression analysis of 34 ribosome-related genes, displaying 18 ribosome-related genes (*p* < 0.05). (**B**) Five gene expression features based on ribosome related-clusters were selected with the LASSO Cox model. (**C**) Tuning parameter selection for ribosome-associated genes in the LASSO model in TCGA BC patients. (**D**,**E**) Kaplan–Meier survival curves showed lower survival rates for high-risk patients than low-risk groups in both the TCGA and GEO cohorts.

To nail down the role of these two subtypes in the clinical treatment of BC, and calculate the risk score for BC patients, we performed univariate COX regression analysis of 34 ribosome-related genes previously obtained, and 18 ribosome related genes were *p* < 0.05 in the TCGA cohort (Figure 9A). The LASSO regression algorithm finally confirmed five ribosome-related genes in Figure 9B,C (*RPS18*, *RPL11*, *RPLR1*, *RPL27A*, and *RPL38*), named ribosome risk score signature (RRS), which will be used for the construction of ribosome signature. Risk score = −0.088 × (*RPS18* expression) + −0.155 × (*RPL11* expression) + −0.024 × (*RPLP1* expression) + −0.153 × (*RPL27A* expression) + −0.047 × (*RPL38* expression). After analyzing the prognostic survival of risk score and TCGA BC patients by using the Kaplan–Meier curve, we found the survival rate of the high-risk group was significantly lower than that of the low-risk group (Figure 9D). The same results were obtained by using the GSE20785 data set to verify (Figure 9E). The results of T-SNE and PCA analysis proved the rationality of the RRS model in the division of both high-risk and low-risk groups in TCGA (Figure S4A,C) and GEO cohort (Figure S4B,D). The risk curve analysis showed that the mortality rate of the low-risk group was lower and the survival time was longer (as shown in the left side of the imaginary line in the graph), while the number and mortality of the high-risk group were positively correlated with the risk score (Figure S4E–H). The 3-year AUCs curve in the TCGA breast cancer patient cohort was 0.568, and the 5-year AUCs curve was 0.587. (Figure S4I). The AUCs curves for 3 and 5 years in the GSE20785 cohort were 0.554 and 0.583, respectively (Figure S4J).

### 3.6. Clinicopathological Characteristics, Immune Activity, and Functional Enrichment Analysis Based on the RRS Model

**Figure 10:**
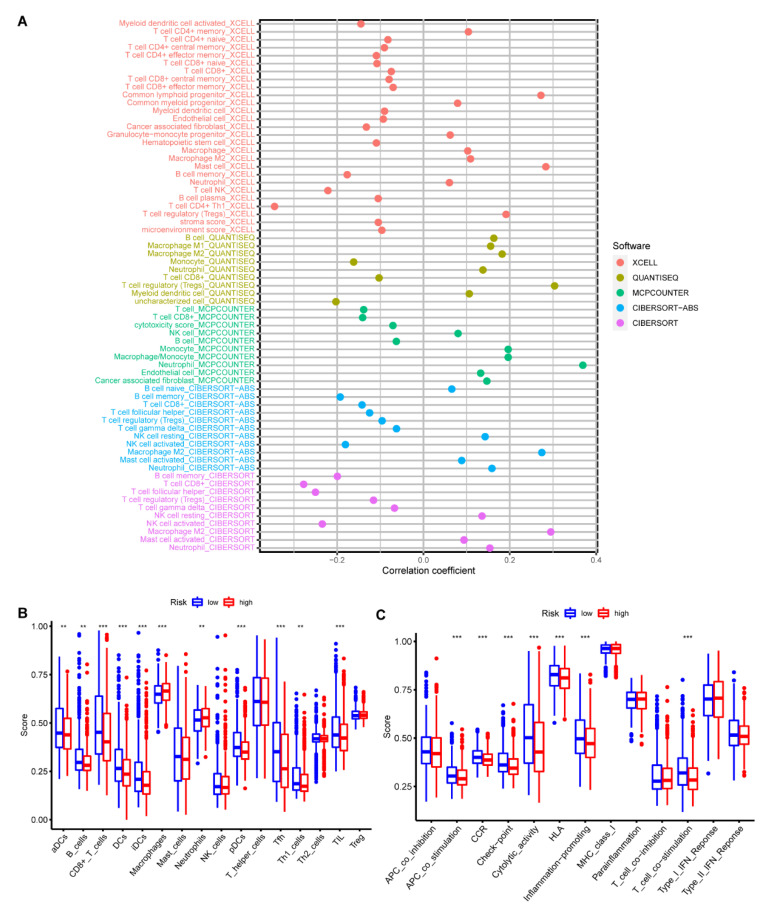
Comparison of ssGSEA scores for immune cells and immune pathways between high- and low-risk groups. (**A**) Differences in immune cell infiltration between high- and low-risk groups. (**B**,**C**) Enrichment scores of 16 immune cells and 13 immune-related pathways between the low- and high-risk groups are shown with box plots in TCGA cohorts. *p* < 0.01 = “**”, and *p* < 0.001 = “***”.

**Figure 11:**
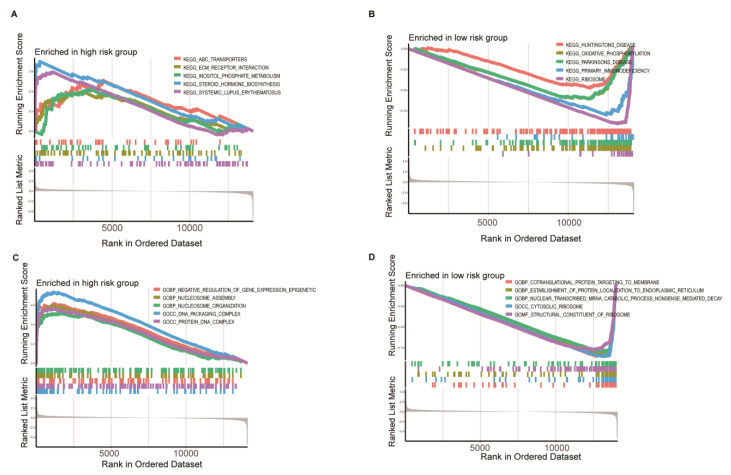
GSEA enrichment analysis was performed according to the risk score of ribosome-associated genes for the high-risk group and low-risk groups in TCGA BC cohorts.(**A**,**B**) KEGG enrichment in high and low risk groups.(**C**,**D**) GO enrichment in high and low risk groups.

The following was a comprehensive analysis of TCGA BC cohort. The results of the heatmap reveal that the RRS model risk score was significantly correlated with age (Figure S5A). The results of univariate and multivariate cox algorithms demonstrate that RRS has an important role in the prognostic assessment of breast cancer patients (Figure S5B,C). XCELL, QUANTISEQ, MCPCOUNTER, CIBERSORT, and CIBERSORT-ABS algorithm results identify that the high-risk group displayed a positive correlation with macrophage M2, while low-risk group showed a favorable correlation with CD8^+^ T cells (Figure 10A). Details of immune cell infiltration in the high- and low-risk groups are shown in Supplementary Table S2. This result is similar to previous results of immune infiltration based on 34 ribosome-associated hub genotypes. For this reason, we compared and analyzed the enrichment fraction of 16 immune cells and the activity of 13 immune-related pathways between the high-risk group and the low-risk group in the RRS model. The infiltration of aDCs, B cells, CD8^+^ T cells, dendritic cells (DCs), induced dendritic cells (iDCs), and plasmacytoid tumor-infiltrating lymphocytes (TILs) in the low-risk group was higher than that in the high-risk group (Figure 11B). However, macrophages and neutrophils showed higher infiltration in the high-risk group than in the low-risk group (Figure 10B). APC co-stimulation, chemokine receptor (CCR), immune checkpoint, cytolytic activity, human leukocyte antigen (HLA), inflammation promotion, and Type1-IFN response activity in low-risk group were well above the high-risk one (Figure 10C). The GSEA algorithm explores the function of KEGG and GO for high- and low-risk groups. The results of KEGG enrichment analysis showed that the main enriched pathways in the high-risk group were ABC transporters, ECM–receptor interaction, inositol phosphate metabolism, steroid hormone biosynthesis, and systemic lupus erythematosus (Figure 11A). The low-risk group pathways were enriched in ribosome, Huntington’s disease, oxidative phosphorylation, Parkinson’s disease, and primary immunodeficiency (Figure 11B). In addition, GO enrichment results in the high-risk group were focused on ribosome assembly, ribosome organization, DNA packaging complexes, and protein DNA complexes. (Figure 11C). Results of the low-risk group revealed a significant correlation with cytosolic ribosome, structural constituents of ribosome, nuclear transcribed mRNA catabolic process nonsense mediated decay, establishment of protein localization to endoplasmic reticulum, and cotranslational protein targeting to membrane (Figure 11D).

## 4. Discussion

Our study conducted a thorough and multi-layered analysis on the alterations in the activity of immunological markers and gene sets in both BC tissue and adjacent tissue, offering new insight into the significance of adjacent tissue in the prognosis of BC instead of solely focusing on changes within BC tissue. Previous studies on BC subtypes and prognosis were still limited to BC itself, and the changes between BC and adjacent tissues were not analyzed as a whole. For example, classification of breast cancer into different subtypes have been based on pyroptosis-related genes, metabolic characteristics, and multi-omics features [5,6,7]. These studies have important significance in the prognosis diagnosis and targeted treatment of BC. However, the adjacent breast tissue cannot be ignored in the process of BC. Therefore, we divided BC patients into three survival-related subtypes with an NMF algorithm after analyzing the immunological and marker gene set activities of BC tissues and adjacent sample tissues. In summary, subtype 2 had the better prognosis compared with subtype 1 and subtype 3, and subtype 3 had the worst prognosis. Additionally, the proportion of immune cells, stromal cells, and enrichment of immune-related pathways in subtype 2 were also higher than those in subtype 1 and subtype 3. Previous research had revealed that the activation of immune cells and immunity pathways in tumor tissues has an essential roles in the prognosis of cancer patients [22,23]. Using the LASSO Cox algorithm, we found a gene set of prognostic genes in BC adjacent tissues, and the results of KEGG and GO enrichment algorithms suggested that the gene set was associated with ribosome and cytoplasmic translation and constitutive of ribosome. Interestingly, through the PPI algorithm and MCODE algorithm, we found that 34 hub genes were also highly correlated with ribosome. The changes in the number and modification of ribosomes can affect the growth and proliferation of tumor cells, suggesting that ribosomes were intensively associated with tumor cells [24]. Our study further confirms our research results and demonstrated the important function of ribosome-associated genes in the process of BC. Next, according to the expression levels of 34 ribosome associated genes, two ribosome associated subtypes were identified.

The survival rate of BC patients in C1 was better than that in C2. CD8^+^ T cells in C1 were also higher than that in C2, and CD8^+^ T cells have a strong tumor-killing effect [25]. The ribosome signature of five ribosome-related genes (*RPS18*, *RPL11*, *RPLP1*, *RPL27A*, and *RPL38*) was determined by using single factor Cox analysis and LASSO analysis, and its accuracy and reliability were verified by the GEO dataset. In the risk model constructed with these five ribosome-related genes, we found that the number of macrophages and neutrophil infiltrates was significantly higher in the high-risk group. The malignant potential of breast cancer may directly influence the infiltration of neutrophils, and the more aggressive triple negative breast cancer (TNBC) is able to attract more neutrophils. They may promote cancer development and metastasis by creating a pro-inflammatory microenvironment [26]. Tumor-associated macrophages have the ability to influence the progression of TNBC by regulating the activity of hepatic leukemia factor (HLF) through secretion of transforming growth factor-beta1 (TGF-β1). This leads to an increase in the proliferation, metastasis, and resistance of TNBC cells [27]. However, the activity of immune checkpoint-related pathways was higher in the low-risk group than in the high-risk group. The level of immune checkpoint activity may not consistently align with the level of breast cancer risk, suggesting the presence of other influencing factors. The main KEGG results in the high-risk group were focused on inositol phosphate metabolism and ECM–receptor interaction. Detection of changes in the activity of the inositol phosphate metabolic pathway in patients with early-stage breast cancer has important clinical implications [28].

The ECM–receptor interaction signaling pathway was also identified as a potential contributor to the development of breast cancer [29]. RPS18 is a reference parameter in the demonstration of multiplex nucleic acid sequence-based amplification (NASBA) on microarray analysis for breast cancer diagnostics [30]. Previous research has demonstrated a correlation between high levels of expression of RPL11 and improved overall survival rates in breast cancer patients. This association has been considered a positive prognostic indicator, and is consistent with our results [31]. RPLP1 can indeed lead to the accumulation of reactive oxygen, which activates the MAPK1/ERK2 signaling pathway and enhances the growth of breast cancer cells [32]. tRNA-derived fragment-19-W4PU732S can target to inhibit RPL27A to accelerate the malignant activity of breast cancer cells [33]. RPL38 can be used as a potential target for immunotherapy of BC [34].

Our research still has some limitations. Firstly, the verification group has less samples. Since there were few arrays containing paired tumor and non-tumor samples of expression and clinical information, we have to choose gene expression profiles that combine multiple different array platforms to further validate our classification. Second, the prognostic gene sets identified in our study has not been validated in our clinical samples. In the future, we will devote more energy to evaluate the prediction effect of the above gene sets multicenter samples and explore their clinical application value. Therefore, further studies will be needed to confirm our results. Our study provides significant assistance to patients after mastectomy, who should be more concerned about changes in ribosome-related gene activity in nontumor tissues adjacent to BC.

## 5. Conclusions

To sum up, three BC immune-related clinical subtypes were established based on changes in immune-related pathway activity in tumor and non-tumor tissues. The biological functions performed by ribosome-related genes in the three subtypes may determine the prognosis of BC patients. RRS can be a biological marker of prognosis for BC patients that might be of some benefit in BC treatment. Changes to ribosome-related genes in non-tumor tissues adjacent to BC samples should be focused upon further during the treatment of BC.

## Data Availability

Data used in our study can be downloaded from TCGA (https://portal.gdc.cancer.gov/) and GEO (https://www.ncbi.nlm.nih.gov/geo/). Visit the website and download the data on 24 November 2021.

## Supplementary Materials

The following supporting information can be downloaded at: https://www.mdpi.com/article/10.3390/medicina59030424/s1, Figure S1: Identification of clinical subtypes associated with BC in GEO; Figure S2: Different phenotypes of BC subtypes in GEO; Figure S3: PPI network revealed the interactions of the N gene set; Figure S4: Validation of the Ribosome related signature (RRS) based on ribosome related clusters in TCGA cohort.; Figure S5: Analysis of risk scores and clinicopathological characteristics; Table S1: Clinical data from TCGA and GEO; Table S2: Description of immune cell infiltration.
