# Supplementary material for: A Ribosome-Related Prognostic Signature of Breast Cancer Subtypes Based on Changes in Breast Cancer Patients’ Immunological Activity
Source: Medicina (Kaunas). 2023 Feb 21;59(3):424. doi: 10.3390/medicina59030424 (PMC10051894; doi:10.3390/medicina59030424)
Supplement: Supplementary file 1 [file medicina-59-00424-s001.zip › Supplementary Figures S1-S5.pdf]

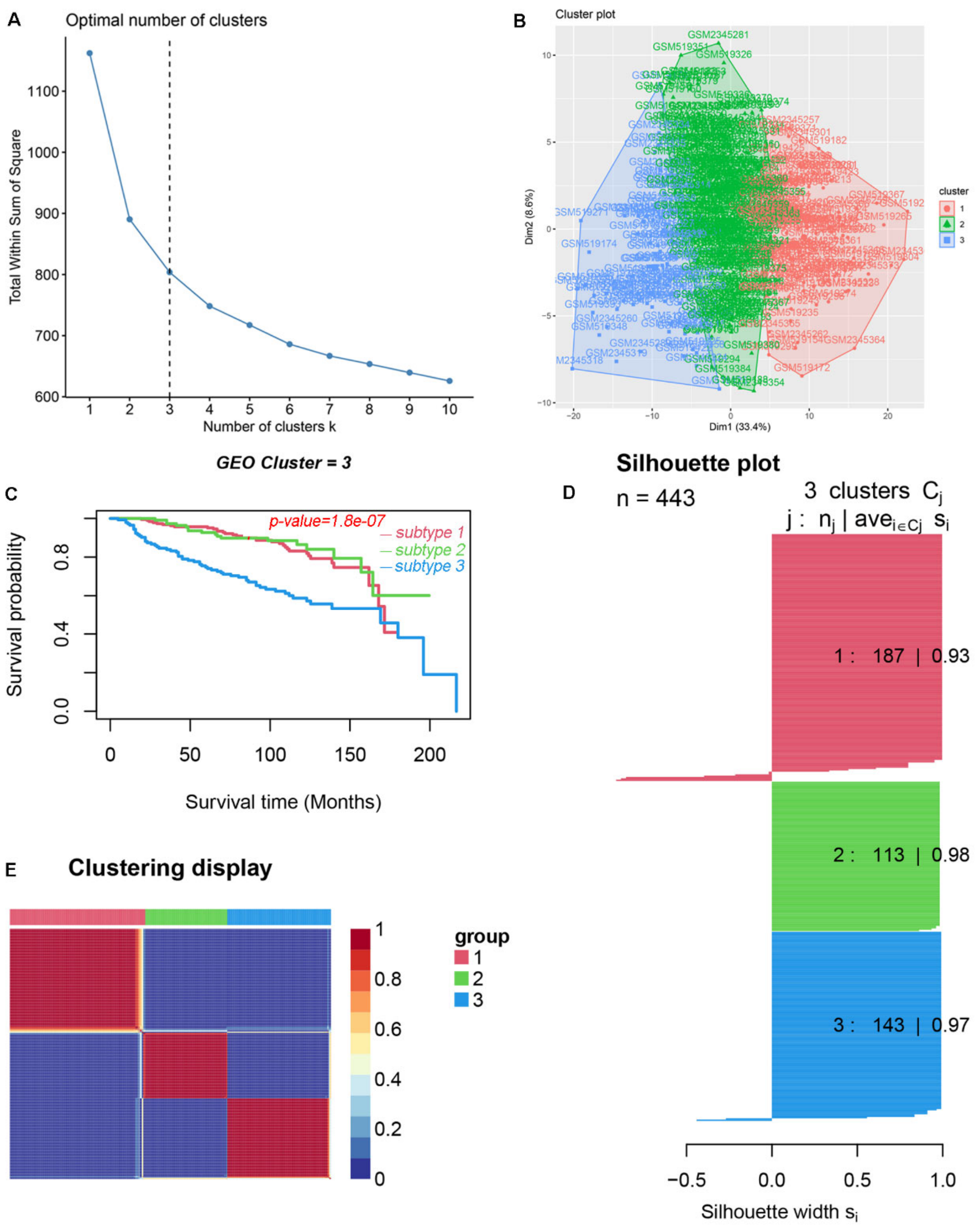

Figure S1. Identification of clinical subtypes associated with BC in GEO.(A)Third, the optimal number of clusters (B)Visualize the cluster results. (C) Kaplan-Meier survival analysis of three BC subtypes. (D) Results of the Silhouette Width plots. (E) Nonnegative matrix decomposition (NMF) was used for cluster analysis of BC samples.

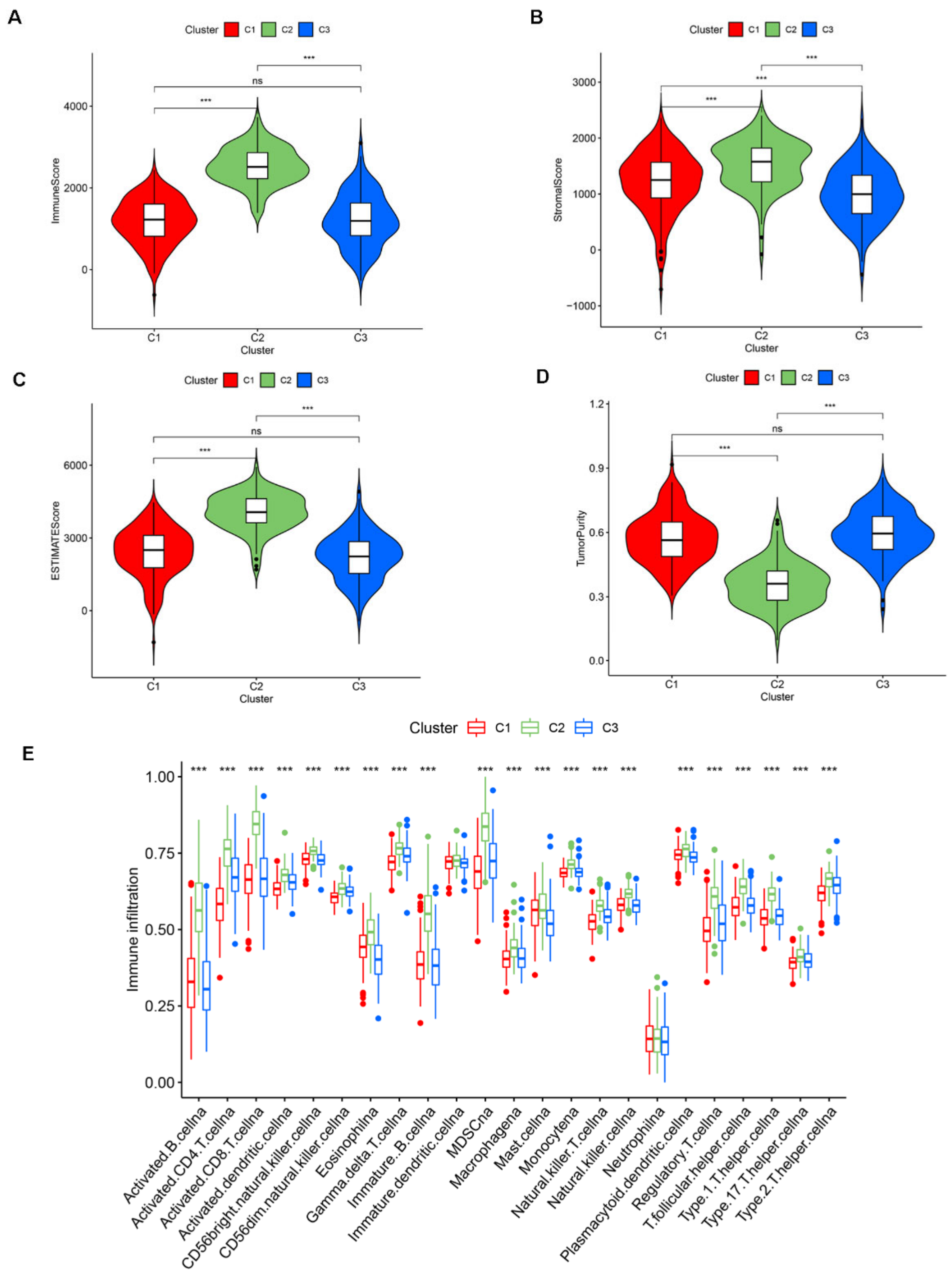

Figure S2. Different phenotypes of BC subtypes in GEO. (A-D) The levels of immune cell infiltration, stromal score, ESTIMATEScore and tumor purity were compared among BC subtypes of GEO. (E) Comparison of the expression levels of 23 kinds of immune cells between BC subtypes in GEO.

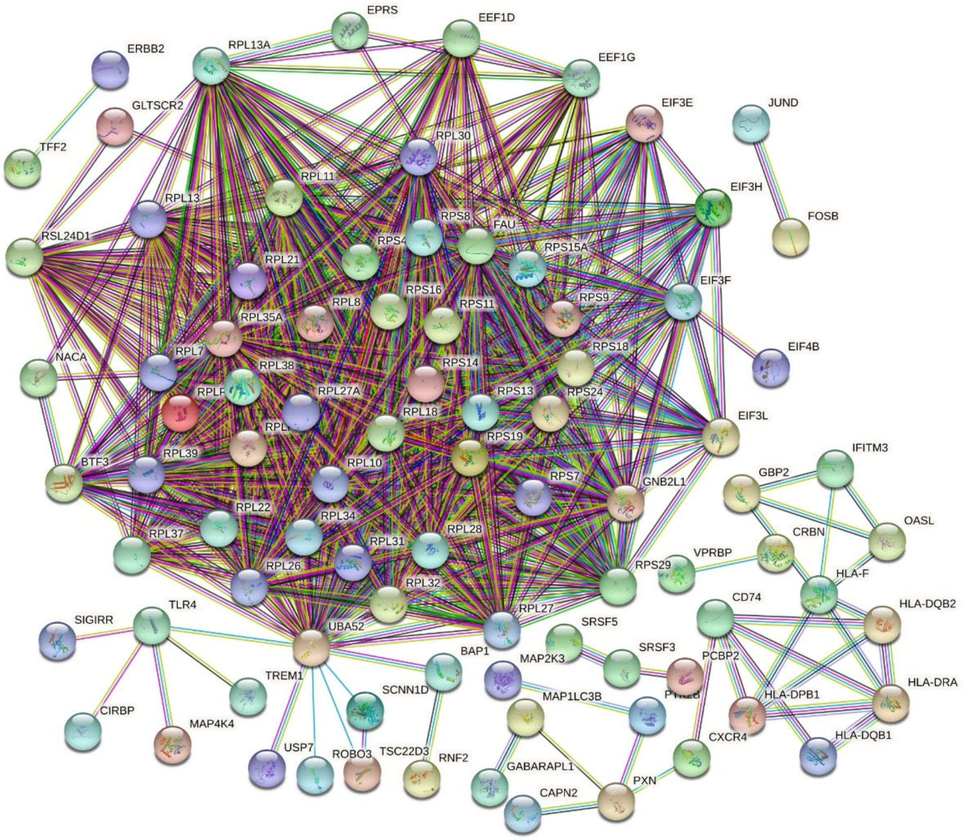

Figure S3. PPI network revealed the interactions of the N gene set (interaction score = 0.9).

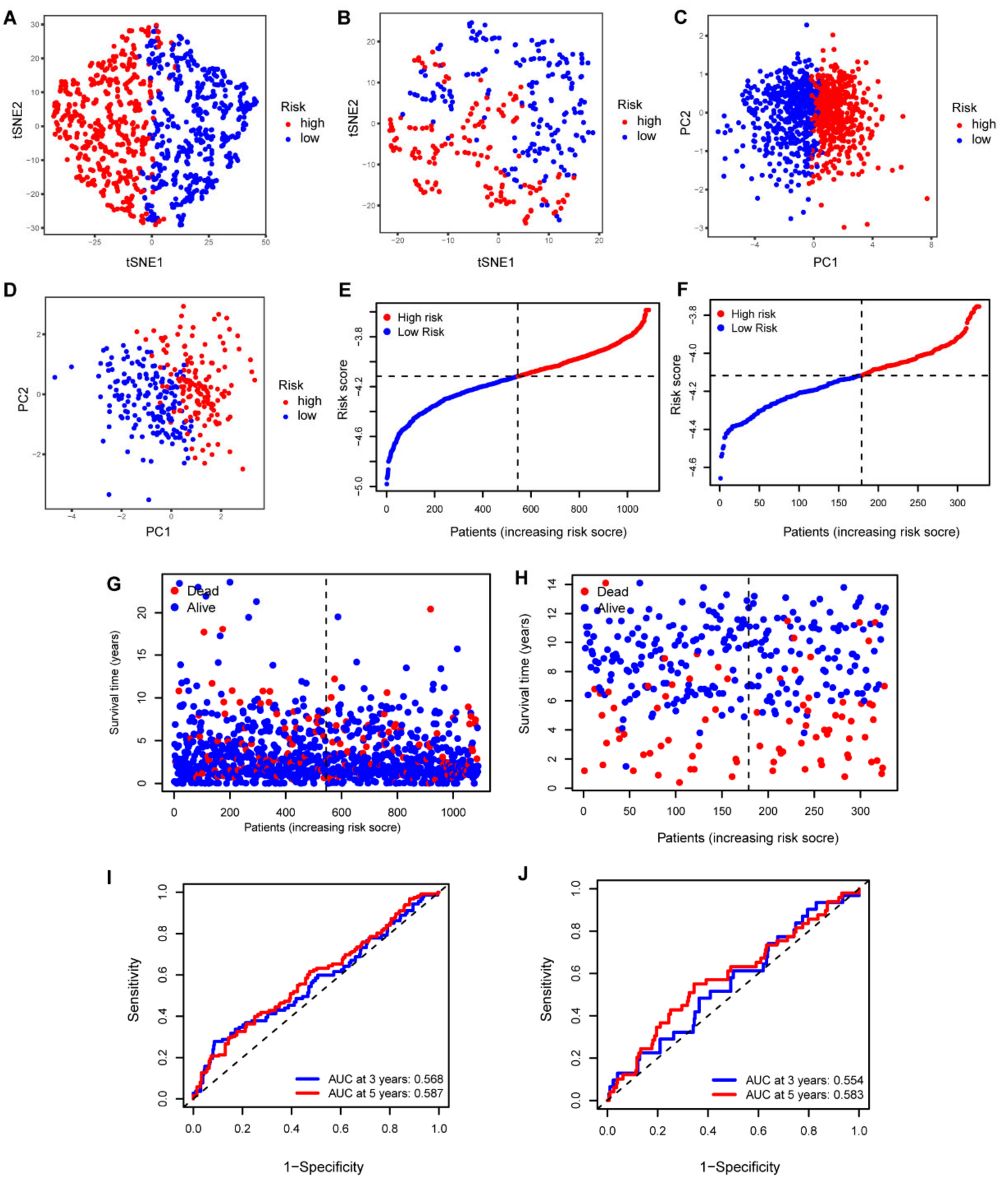

Figure S4. Validation of the Ribosome related signature (RRS) based on ribosome related clusters in TCGA cohort.

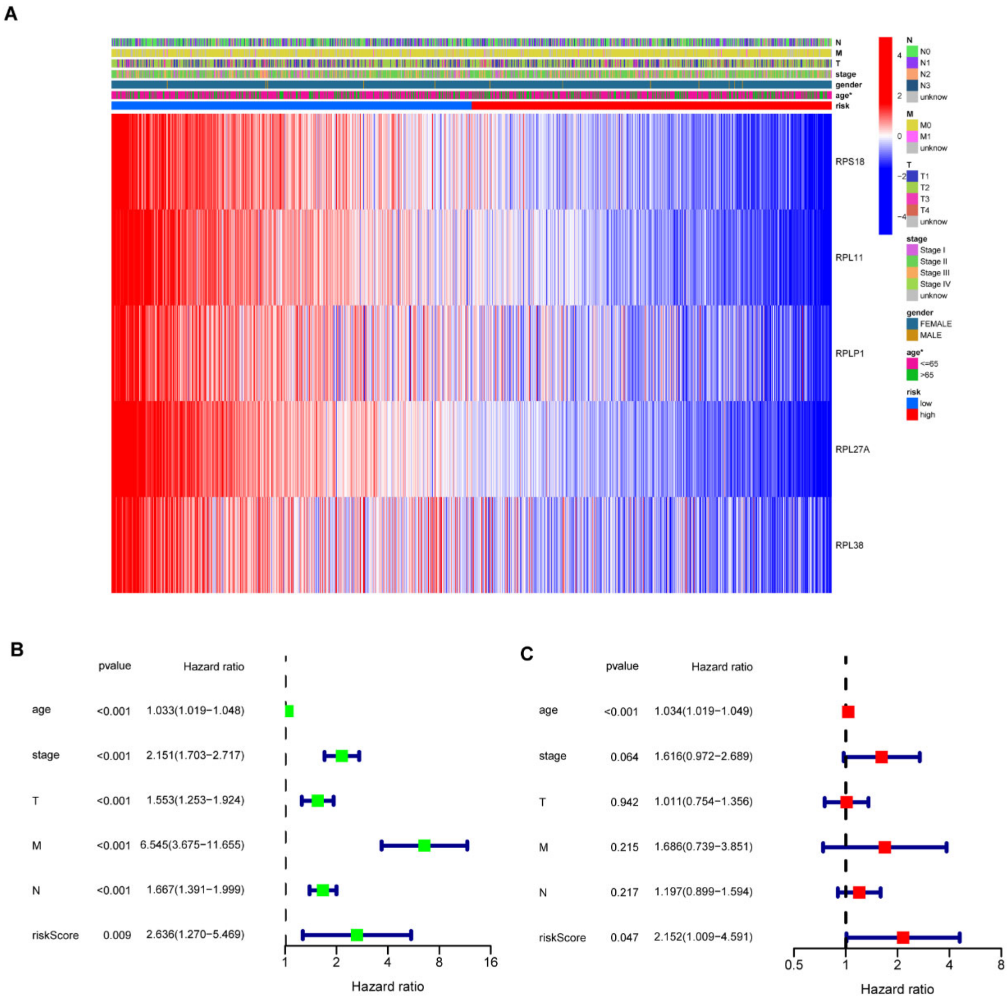

Figure S5. Analysis of risk scores and clinicopathological characteristics(A) Heatmap showing correlation of risk score-related genes with clinicopathological features (B, C) Univariate and multivariate Cox regression analyses revealed that the risk score for ribosome related genes in TCGA BC cohort.
